# A systematic review of maternal smoking during pregnancy and fetal measurements with meta-analysis

**DOI:** 10.1371/journal.pone.0170946

**Published:** 2017-02-23

**Authors:** Miriam Abraham, Salem Alramadhan, Carmen Iniguez, Liesbeth Duijts, Vincent W. V. Jaddoe, Herman T. Den Dekker, Sarah Crozier, Keith M. Godfrey, Peter Hindmarsh, Torstein Vik, Geir W. Jacobsen, Wojciech Hanke, Wojciech Sobala, Graham Devereux, Steve Turner

**Affiliations:** 1 Child Health, University of Aberdeen, Aberdeen, United Kingdom; 2 FISABIO – Universitat Jaume I – Universitat de València Epidemiology and Environmental Health Joint Research Unit and Spanish Consortium for Research on Epidemiology and Public Health (CIBERESP), Valencia, Spain; 3 The Generation R Study, Department of Paediatrics, Department of Epidemiology, Erasmus MC, University Medical Centre Rotterdam, Rotterdam, The Netherlands; 4 MRC Lifecourse Epidemiology Unit and NIHR Southampton Biomedical Research Centre, University of Southampton and University Hospital Southampton NHS Foundation Trust, Southampton, United Kingdom; 5 University College London, London, United Kingdom; 6 Faculty of Medicine, Norwegian University of Science and Technology, Trondheim, Norway; 7 Department of Environmental Epidemiology, Nofer Institute of Occupational Medicine, Lodz, Poland; Legacy, Schroeder Institute for Tobacco Research and Policy Studies, UNITED STATES

## Abstract

**Background:**

Maternal smoking during pregnancy is linked to reduced birth weight but the gestation at onset of this relationship is not certain. We present a systematic review of the literature describing associations between maternal smoking during pregnancy and ultrasound measurements of fetal size, together with an accompanying meta-analysis.

**Methods:**

Studies were selected from electronic databases (OVID, EMBASE and Google Scholar) that examined associations between maternal smoking or smoke exposure and antenatal fetal ultrasound measurements. Outcome measures were first, second or third trimester fetal measurements.

**Results:**

There were 284 abstracts identified, 16 papers were included in the review and the meta-analysis included data from eight populations. Maternal smoking was associated with reduced second trimester head size (mean reduction 0.09 standard deviation (SD) [95% CI 0.01, 0.16]) and femur length (0.06 [0.01, 0.10]) and reduced third trimester head size (0.18 SD [0.13, 0.23]), femur length (0.27 SD [0.21, 0.32]) and estimated fetal weight (0.18 SD [0.11, 0.24]). Higher maternal cigarette consumption was associated with a lower z score for head size in the second (mean difference 0.09 SD [0, 0.19]) and third (0.15 SD [0.03, 0.26]) trimesters compared to lower consumption. Fetal measurements were not reduced for those whose mothers quit before or after becoming pregnant compared to mothers who had never smoked.

**Conclusions:**

Maternal smoking during pregnancy is associated with reduced fetal measurements after the first trimester, particularly reduced head size and femur length. These effects may be attenuated if mothers quit or reduce cigarette consumption during pregnancy.

## Introduction

Maternal smoking during pregnancy is associated with a reduction in birth weight of approximately 250g and is known to adversely affect the health of both fetus and mother.[1] Knowledge of the age at onset of faltering fetal growth in association with maternal smoking would be useful evidence to underpin public health advice for mothers not to smoke during pregnancy. The advent of ultrasound in the mid-1980s provided an opportunity to study antenatal fetal size and growth as indices of fetal wellbeing, and there is now a rapidly expanding literature of “fetal epidemiology”.

The literature describing associations between maternal smoking and reduced fetal measurements is inconsistent. For example, maternal smoking is associated with reduced second trimester growth in some studies[2,3] but not all[4] and abdominal and proximal muscle growth restriction has been linked to maternal smoking in one population[5] but to peripheral fetal growth (i.e. femur length) in others.[2,3] One study reported an association between exposure to maternal smoking and increased fetal head and arm growth.[6] Here we report a systematic review of the literature with meta-analysis to answer the question “at which gestational ages are the associations between exposure to maternal smoking apparent?” In secondary analyses we sought to relate fetal size to high or low cigarette consumption and the cessation of smoking before or during pregnancy.

## Materials and methods

### Rationale, inclusion criteria and search strategy

We have previously completed a systematic review linking antenatal size and growth to risk for postnatal outcomes linked to non-communicable diseases[7] and then sought to identify which potentially modifiable environmental exposures were linked to reduced fetal size. A database search was carried out in August 2014 and updated in May 2016 using OVID MEDLINE, EMBASE and CINAHL databases. Studies where fetal ultrasound anthropometric measurements related to maternal environmental exposures were included. Papers which related maternal exposures to congenital malformations (e.g. renal cysts) were excluded. The number and diversity of papers identified persuaded us to present the maternal smoking literature separately from other exposures. Search terms were identified after reviewing relevant publications already known to the authors from our earlier work[7] and are displayed in S1 Fig. Papers were also identified from the following cohorts known to have fetal measurement data: the Raine cohort; (http://www.rainestudy.org.au/); the EDEN cohort (https://eden.vjf.inserm.fr); Southampton Women Survey (SWS, http://www.leu.soton.ac.uk/sws/); the Generation R study (http://www.erasmusmc.nl/epi/research/Generation-R/); and INMA Mother and Child Cohort Study[8]. References of identified papers were also searched to identify additional studies. Abstracts were independently reviewed by two authors and studies which fell within our predefined inclusion criteria were identified and full papers obtained. Ethics approval was not required for this systematic review and meta-analysis since no patient contact took place.

### Fetal measurements

The measurements considered in the first trimester (i.e. ≤13 weeks gestation) were: crown rump length (CRL), biparietal diameter (BPD), head circumference (HC) abdominal circumference (AC) and mean abdominal diameter (MAD). For the second trimester (i.e. 14 to <28 weeks gestation) and third trimester (i.e. ≥28 weeks) femur length (FL), HC, BPD, MAD, AC and estimated fetal weight (EFW) were considered.

### Data synthesis for meta-analysis

Standardised fetal measurements were derived for each fetal measurement for every population. No interaction terms were sought since our focus was on the relationship between maternal smoking and fetal size. Original data were provided by the custodians of three datasets (Scand_SGA[4,9,10], Prabhu *et al*[3] and Pringle *et al*[11]) where EFW was derived[12] and Z scores for fetal measurements calculated using linear regression which considered sex, gestation and maternal height. Previously unpublished data were also provided in a format for meta-analysis by four other cohorts ([2,13,14] and SWS). Results from one published study[15] were also included. Mean absolute and standardised fetal measurements were reported since this paper[15] reported only absolute measurements. Where more than one publication arose from a single cohort (e.g. Scand-SGA[4,9,10]), a single dataset was used to derive results for meta-analysis. Z scores (but not absolute values) of MAD and HC were considered as interchangeable measurements for AC and BPD respectively in the meta-analysis. Review Manager (version 5.3.5) was used for meta-analysis. The following secondary analyses were carried out: (i) comparison of fetal measurements between individuals exposed to lower or higher maternal cigarette consumption (ii) comparison of fetal measurements for individuals whose mothers quit before becoming pregnant and never smokers and (iii) comparison of fetal measurements for individuals whose mothers quit during pregnancy and never smokers. The secondary analyses were restricted to those fetal measurement where maternal smoking (yes/no) was associated with altered z scores of fetal measurements. The risk of bias and heterogeneity were explored using funnel plots and I^2^ (for the latter, a value of >50% was considered indicative of substantial heterogeneity[16]). The Effective Public Health Practice Project tool was used to assess the quality of the studies included in the final review (http://www.ephpp.ca/PDF/Quality%20Assessment%20Tool_2010_2.pdf).

## Results

### Study selection

**Fig 1:**
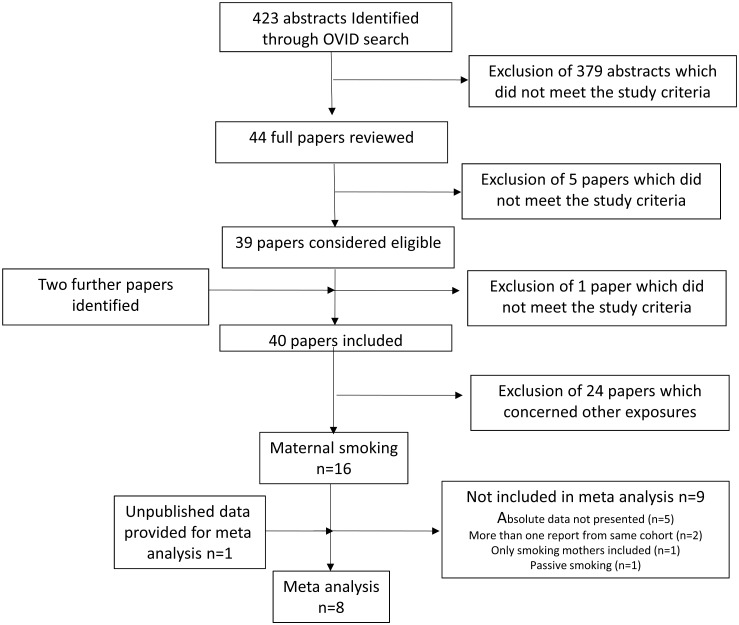
Flow style diagram showing how the papers included in the review were identified.

The search identified 284 abstracts and 16 papers were included in this review,[2–6,9–11,13–15,17–21] Fig 1. Studies were excluded that related maternal smoking to fetal organ volumes including total lung and renal volumes[22,23], brain[24] or kidney[25]) and abdominal fat.[26] One paper were identified from reading reference lists[9]. One publication[27] was considered but excluded since the proportion of maternal smoking was very low (2.2%).

### Study characteristics

There was one small randomised controlled study,[20] two case-control studies,[5,28] one case only study[18] and the remainder were prospective cohorts. One paper compared maternal cotinine to fetal measurements[14] and the remainder relied upon maternal reported smoking. There were three studies with strong study design,[9,14,17] nine with moderate and four with weak study design;[4,15,18,21]. For each study included, Table 1 describes the quality of study design, the direction of any association and in which trimester any association was present. See S1 Table for full results of the quality control.

**Table 1:** Summary of results from the papers identified in the systematic review.

	Number of pregnant mothers recruited	Study design 1 = strong, 2 = moderate, 3 = weak	Second trimester				Third trimester			
			HC/ BPD	FL	AC/ MAD	EFW	HC/ BPD	FL	AC/MAD	EFW
Jeanty 1987 [21]	952	3					X	X		
Newnham 1990[15]	535	3	↓	X	↓		↓	X	↓	
Vik 1996[9]†	530 (185 smokers)	1	X	X	X		X	↓	↓	
Bernstein 2000[5]	101 (65 smokers)	2							↓	↓
Zaren 2000[10]†	865 (550 smokers)	2	X	X	↓		↓	X	↓	
Lampl 2003[6]	366 (87)	2	↑	X	↑		↑	↓		
Hanke 2004[14]*	183 (25 smokers)	1	↓	X	X					
Pringle 2005[11]	1650 (347 smokers)	2	X	X	X		X	↓	↓	
Jaddoe 2007[2]	7098 (1809 smokers)	2	X	↓	X		↓	↓	↓	
Bergsjo 2007[4]†	561 (170 smokers)	3	X	X	X					
Veilwerth 2007[18]	269 (all smokers)	3						X		↓
Heil 2008[20]	77 (all smokers)	2								↓
Prabhu 2010[3]	1210 (375 smokers)	2	X	↓						
Iniguez 2012[19]*‡	780 (319 smokers)	2	X	X	X	X	↓	↓	↓	↓
Lindell 2012[17]	56792 (5822 smokers)	1								↓
Iniguez 2013[13]‡	2478 (788 smokers)	2	X	X	X	X	↓	↓	↓	↓

HC = head circumference, BPD = biparietal diameter, AC = abdominal circumference, MAD = mean abdominal circumference, EFW = estimated fetal weight. X = no association present, ↑exposure associated with increased fetal measurement, ↓ exposure associated with reduced fetal measurement and empty boxes indicate that the outcome was not reported.

*maternal active and passive smoke exposure reported.

^†^reports from the SGA_SCAND study.

^‡^reports form the INMA cohort

### Systematic review

#### Fetal size and maternal smoking

Three publications were based on one cohort[4,9,10] and two publications on a second cohort[13,19] and results are summarised for each paper. Maternal smoking was not associated with altered first trimester size in the two studies identified.[3,13] The relationship between maternal smoking and second trimester fetal measurements differed between studies: BPD was reduced in association with maternal smoking in two papers,[14,15] was increased in a third[21] and not changed in eight [2–4,9–11,13,19]; FL was reduced in association with maternal smoking in two papers[2,3] but not in nine [4,6,9–11,13–15,19]; and AC or MAD was reduced in association with maternal smoking in two studies,[10,15] increased in one[6] and not changed in seven.[2,9,11,13,14,19] In the third trimester, maternal smoking was associated with reduced EFW and AC (or MAD) in all studies reporting these measurements. Third trimester BPD or HC was reduced in fetuses whose mothers smoked in five studies,[2,10,13,15,19] BPD was increased in one study[6] and not associated with maternal smoking in three.[9,11,21] Third trimester femur length was reduced in six studies [2,6,11,13,19] and not associated with maternal smoking in four studies.[10,15,18,21] Fuller details are presented in S2 Table.

#### Fetal growth and maternal smoking

Maternal smoking was associated with reduced growth in the second or third trimester in all six studies identified.[2,5,6,13,17,20] The studies reported different growth outcomes making meta-analysis impractical, but the magnitude of association between maternal smoking and fetal size differed between studies: maternal smoking was associated with (i) a reduction of 8–10% of standard deviation score for growth in EFW, FL and BPD between 20 and 34 weeks gestation; [13] (ii) a mean reduction in EFW between 33 weeks and term of 0.13 z score;[17] (iii) a reduction in HC and AC growth equivalent to 0.5mm/week between 13 and 30 weeks gestation[2] or reduced AC growth by ~1mm/week between 27 and 37 weeks gestation[5]. Abstinence from smoking was associated with increased growth in EFW, FL and AC (approximately 50g/week, 0.3 and 2 mm/week respectively) between 30 and 34 weeks gestation compared to ongoing smoking.[20]

#### Fetal size and passive smoke exposure

Among non-smoking mothers, exposure to second hand smoke exposure in restaurants (but not home or workplace) was associated with reduced BPD between 20 and 32 weeks but not 32–38 weeks.[19] When passive maternal exposure to tobacco smoke was defined as <10ng/mL plasma cotinine, there was a negative association which approached significance between plasma cotinine and second trimester BPD.[14]

### Meta-analysis

The details available from the eight populations included in the meta-analysis are presented in Table 2.

**Table 2:** Details available from the studies used in the meta-analysis of the association between maternal smoking status and fetal ultrasound measurements.

	First trimester data available		Second trimester data available		Third trimester data available		Smoking categories compared	Other comments
	Absolute measurement	Z score	Absolute measurement	Z score	Absolute measurement	Z score		
Newnham 1990[15]			√ (AC, BPD and FL at 18 weeks)		√ (AC, BPD and FL at 34 weeks)		No	Smoking status ascertained at each ultrasound assessment. Identifies ex-smokers. Data presented separately by gender, data for boys used for meta-analysis. Number exposed to smokers >20 cpd very small and comparison between smoking categories was not carried out
Hanke 2004[14]			√ (AC, BPD and FL at 22 weeks)	√			No	Smoking status determined once (at recruitment). Identifies passive smoke exposed. Z score adjusted for gestation
Pringle 2005[11]	√ (CRL and FL 13 weeks)	√	√ (AC, BPD, EFW and FL at 20 weeks)	√	√ (AC, BPD, EFW and FL at 32 weeks)	√	1–9 vs 10–20 cpd	Smoking status determined once (at recruitment). Identifies quit during pregnancy. Z score adjusted for sex, maternal height, gestation.
Jaddoe 2006[2]	√ (HC and FL at 13 weeks)	√	√ (AC, HC, EFW and FL at 21 weeks)	√	√ (AC, HC and FL at 30 weeks)	√	1–5 vs ≥10 cpd	Smoking status determined in each trimester. Identifies quit during pregnancy. Z score adjusted for gestation
Prabhu 2010[3]	√ (CRL 11 weeks)	√	√ (AC, BPD, EFW and FL at 20 weeks)	√			1–9 vs ≥10 cpd	Smoking status determined in the first and third trimesters. Identifies quit during pregnancy and ex smokers. Z score adjusted for sex, maternal height, gestation
Iniguez 2013[13]	√ (EFW, BPD, AC and FL at 12 weeks)	√	√ (AC, BPD, EFW and FL at 20 weeks)	√	√ (AC, BPD and FL at 32 weeks)	√	No	Smoking status determined once (32 weeks gestation) Identifies ex smokers. Z score adjusted for sex, maternal height, gestation
SWS	√ (AC and HC at 11 weeks)	√	√ (AC, HC and FL at 19 weeks)	√	√ (AC, HC and FL at 34 weeks)	√	No	Smoking status determined once (at recruitment). Z score adjusted for gestation, sex, maternal age, education, height and BMI
Scand_SGA			√ (MAD, BPD, EFW and FL at 18 weeks)	√	√ (MAD, BPD, EFW and FL at 33 weeks)	√	1–9 vs 10–19 cpd	Smoking status determined once (at recruitment). Z score adjusted for sex, and gestation

SWS = Southampton Women Survey. cpd = cigarettes per day. BPD = biparietal diameter, HC = head circumference, FL = femur length, AC = abdominal circumference, MAD = mean abdominal diameter, EFW = estimated fetal weight.

√ = fetal measurement available.

#### First trimester size

**Fig 2:**
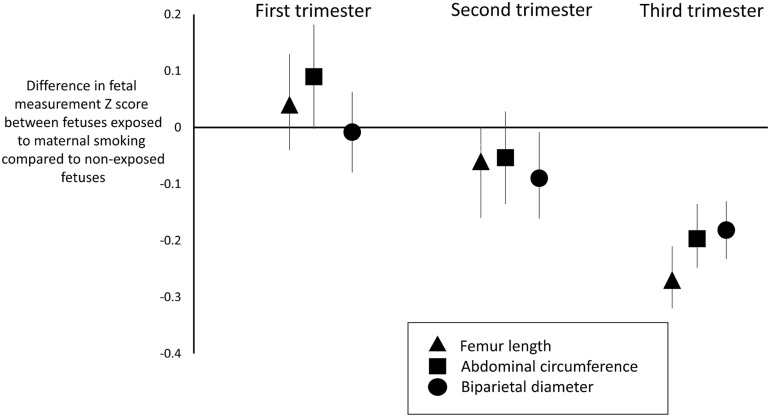
Mean z scores for femur length, abdominal circumference and biparietal diameter for fetuses exposed to maternal smoking relative to non-exposed fetuses. The vertical lines correspond to 95% confidence intervals.

Among two cohorts, there was increased AC z score (mean increased 0.09 z score [95% CI 0.00, 0.18], p = 0.04) for fetuses exposed to maternal smoking compared to unexposed fetuses, Table 3 and Fig 2. No other fetal measurement was associated with maternal smoking, Table 3.

**Table 3:** Summary of meta-analyses relating maternal smoking to absolute and standardised second and third trimester fetal measurements.

	First trimester		Second trimester		Third trimester	
	Absolute difference	Z score difference	Absolute difference	Z score difference	Absolute difference	Z score difference
Biparietal Diameter (or Head Circumference for Z scores)	¶	-0.01 [-0.08, 0.06] 3 cohorts n = 7,423	-0.23mm [-0.59, 0.14] 6 cohorts n = 7,165	-0.09 [-0.16, -0.01]† 7 cohorts n = 14,522	-0.87mm [-1.53, -0.20] ‡4 cohorts n = 4,614	-0.18 [-0.23, -0.13] ‡ 5 cohorts n = 12,195
Femur Length	-0.02 mm [-0.28, 0.23] 2 cohorts n = 4,808	0.04 [-0.04, 0.13] 2 cohorts n = 5,248	-0.22mm [-0.35, -0.10]‡ 8 cohorts n = 15,101	-0.06 [-0.16, -0.01]† 7 cohorts n = 14,730	-1.04mm [-1.44, -0.63] ‡ 6 cohorts n = 12,645	-0.27 [-0.32, -0.21] ‡ 5 cohorts n = 12,490
Abdominal Circumference (or Mean Abdominal Diameter for z scores)	-0.33mm [-1.10, 0.44] 2 cohorts n = 3,307	0.09 [0.00, 0.18]† 2 cohorts n = 3,516	-0.71mm [-3.14, 1.72] 7 cohorts n = 12,086	-0.05 [-0.13, 0.03] 7 cohorts n = 13,092	-3.58mm [-4.54, -2.63] ‡ 5 cohorts n = 11,294	-0.19 [-0.24, -0.13] ‡ 5 cohorts n = 12,505
Estimated Fetal Weight	*	*	-2.01g [-6.92, 2.89] 5 cohorts n = 10,730	-0.03 [-0.08, 0.03] 5 cohorts n = 10,583	-66.2g [-112.9, -19.5] ‡ 4 cohorts n = 9,370	-0.18 [-0.24, -0.11] ‡ 4 cohorts n = 9,471
Crown Rump Length	-1.24mm [-2.74, 0.25] 2 cohorts n = 2,056	-0.08 [-0.19, 0.03] 2 cohorts n = 1,861	*	*	*	*

Values are presented as value for fetuses exposed to maternal smoking relative to unexposed fetuses.

*not measured at this gestation,

^†^p≤0.05,

^‡^p<0.001

^¶^ BPD only measured in one cohort.

#### Second trimester size

**Fig 3:**
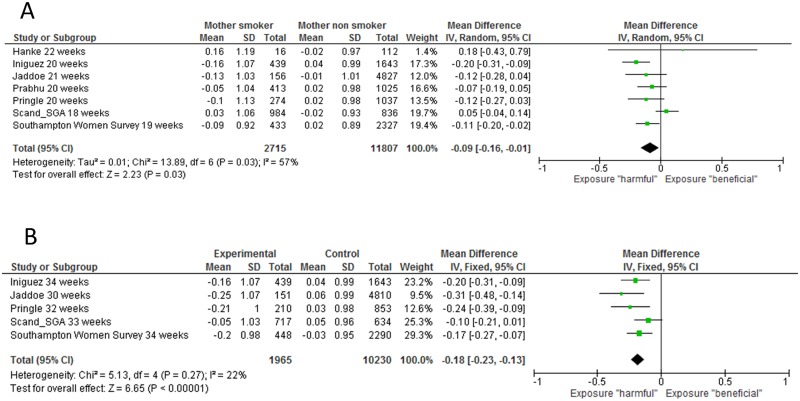
Forest plots demonstrating the association between maternal smoking and fetal head size (biparietal diameter or head circumference) in the second trimester (panel A) or the third trimester (panel B).

Maternal smoking was associated with reduced absolute (p = 0.001) and z score (p = 0.04) FL, Table 3 and Fig 2. Maternal smoking was associated with reduced BPD/HC z score (p = 0.03), Figs 2 and 3, but not with EFW or AC, Table 3. The I^2^ values for heterogeneity between studies exceeded 50% for all absolute fetal measurements except FL and also for standardised measurements for BPD and AC. S2 Fig present forest plots for comparisons of standardised measurement and S3 Fig presents the differences in absolute measurements between fetuses whose mothers did and did not smoke. S4 Fig shows funnel plots for estimates of second trimester standardised scores.

#### Third trimester size

Maternal smoking was associated with reduced absolute and z score values for all fetal measurements, Table 3, Figs 2 and 3. The results were unchanged when z scores for MAD (from the Scand_SGA cohort) were removed from the AC analysis and when z scores for HC results were removed from the BPD analysis (SWS data and Generation R^2^), data not presented. The heterogeneity test across studies was significant for all absolute measurements but for none of the standardised measurements. S5 Fig presents forest plots and I^2^ values comparisons of z scores and between fetuses whose mothers did and not smoke and S6 Fig present differences in the absolute fetal measurements.

#### Secondary analyses

Comparisons between individuals with high relative to low maternal cigarette consumption demonstrated a borderline significant reduction in second trimester BPD z score for the high compared to the low consumption group (four populations, mean reduction 0.09 [0, 0.19] p = 0.05) (S3 Table) and also for third trimester BPD z score (three populations, mean reduction 0.15 [0.03, 0.26] p = 0.01) and FL z score (three populations, mean reduction 0.17 [0.06, 0.28] p = 0.003), S4 Table. There was no reduction in second or third trimester fetal measurements for individuals whose mothers quit during pregnancy compared to non-smokers, S3 and S4 Tables. There was no difference between second trimester measurements for those whose mothers had quit before becoming pregnant and whose mothers had never smoked.

## Discussion

This systematic review of the literature and meta-analysis were designed to describe the gestation at which exposure to maternal smoking became associated with reduced antenatal fetal size and growth. Biparietal diameter and femur length were reduced by at least 0.06 standard deviations (SD) by the second trimester, and all fetal measurements were reduced in the third trimester, typically by 0.2 SD. The reductions in fetal size associated with maternal smoking are statistically significant but small. In the studies where data were available, we also observed an exposure-response relationship for maternal cigarette consumption and reduced second and third trimester fetal head size, and we saw no evidence of reduced measurements among fetuses whose mothers quit before or after becoming pregnant compared to non-smokers. Collectively our findings support current public health advice that mothers should quit whilst pregnant[29] and also suggest that harm reduction might be achieved by reduced cigarette consumption and this now needs exploring in longitudinal studies.

Heterogeneity between studies is not unexpected given the different methodologies used, and in particular some different covariates were included when z scores were derived, but the direction of effect of maternal smoking was consistent although the magnitude did vary. The I^2^ values for standardised fetal measurements indicated little/moderate heterogeneity was likely,[16] whereas those for absolute measurements indicated the presence of considerable/substantial heterogeneity.[16] Heterogeneity for the differences in absolute fetal measurements associated with maternal smoking between studies was most likely explained by not considering covariates, especially gestational age. Funnel plots for second trimester standardised measurements demonstrated heterogeneity for BPD and AC but, acknowledging that there were fewer than the ideal number of ten studies included in the analysis,[30] the plots appeared symmetrical and suggested no obvious bias.

Strength of this work are its novelty and the use of unpublished data from many cohorts for meta-analysis. Among the cohorts included, the fewest number of confounders were included when adjusting fetal measurements from the Generation R study and the greatest for SWS and the reduction in second trimester femur length SD score was greater for the Generation R study compared to SWS (see S2 Fig) and it is possible that consideration of more variables would yield a less obvious association between maternal smoking and fetal femur length in the Generation R study participants. However, it is notable that the absolute femur length measurements were considerably shorter for the Generation R study participants compared to SWS so a corresponding discrepancy in standardised measurements is not unexpected. Furthermore, two cohorts included in the meta-analysis have already described differences in absolute femur length between exposed and unexposed fetuses after adjustment for a comprehensive list of variables.[2,3]

The fetus has traditionally been thought to have a privileged position where it was protected from the adverse effects of environmental exposures by the maternal-placental “unit”, but it is clear that maternal smoking affects fetal wellbeing and growth. Whilst a study such as ours cannot prove causation (although the trial of Heil *et al*[20] points to causation), our results fulfil many of the Bradford Hill[31] criteria including strength, consistency, temporality and biological gradient. Our secondary analyses showed that cessation before and shortly after becoming pregnant was not associated with reduced fetal size and this suggests that the mechanisms affecting fetal growth are predominantly acting in the second half of pregnancy and not before or during early pregnancy. Although maternal smoking may be causally related to small fetal size, other factors such as maternal diet, alcohol and physical activity might be linked to both fetal growth and smoking and partly explain the associations we have observed.

This systematic review has some limitations. First maternal smoking during pregnancy is known to be underreported by mothers[32], and among the studies we considered maternal smoking was only objectively measured in one study[14], thus the magnitude of the association between maternal smoking and small fetal size is likely to be underestimated. Second, the results were restricted to observations made in Western populations and thus may not be generalisable to all populations. Third, secondary analyses were restricted to a subset of populations and it is possible that some of the comparisons would have achieved significance with a larger sample size. Fourth, first trimester dimensions are technically difficult to measure and the absence of association with maternal smoking may at least partly reflect reduced accuracy of measurements, and additionally first trimester data were only available from two cohorts, in one of whom reduced first trimester crown rump length has been reported for fetuses whose mothers who both smoke and do not take folic acid.[33] Finally, data were not available to link maternal smoking in specific trimesters to fetal size in each trimester.

In summary, maternal smoking during pregnancy is associated with reduced fetal size and growth from the second trimester. The relationship between maternal exposure to second hand smoke should be further explored since this exposure is associated with reduced birth weight.[34]

A PRISMA checklist is available as a supporting information (S1 Checklist). The page numbers described in this checklist correspond to page number on the manuscript as submitted and not the manuscript as published.

## Supporting information

S1 Table Quality control analysis for the studies included in this review. (DOCX)

S2 Table A summary of data in each of the studies included in this systematic review. (DOC)

S3 Table Results of the sensitivity analysis for second trimester measurements. (DOCX)

S4 Table Results of the sensitivity analyses for the third trimester. *Data only available in one study.
(DOCX)

S1 Fig A “print screen” showing details of the OVID literature search used in May 2016. (DOCX)

S2 Fig Forest plots showing differences in standardised second trimester measurements between individuals whose mothers smoked and did not smoke. (DOCX)

S3 Fig Forest plots showing differences in absolute second trimester measurements between individuals whose mothers smoked and did not smoke. (DOCX)

S4 Fig Funnel plots for standardised second trimester measurements between individuals whose mothers smoked and did not smoke. (DOCX)

S5 Fig Forest plots showing differences in standardised third trimester measurements between individuals whose mothers smoked and did not smoke. (DOCX)

S6 Fig Forest plots showing differences in absolute third trimester measurements between individuals whose mothers smoked and did not smoke. (DOCX)

S1 PRISMA Checklist (DOC)

## References

[1] Tobacco Advisory Group of the Royal College of Physicians. Passive smoking and children. 2010(ISBN 978-1-86016-376-0).

[2] Jaddoe VW, Verburg BO, de Ridder MA, Hofman A, Mackenbach JP, Moll HA, et al Maternal smoking and fetal growth characteristics in different periods of pregnancy: The generation R study. *Am J Epidemiol*. 2007;165:1207–1215. 10.1093/aje/kwm014 17329715

[3] Prabhu N, Smith N, Campbell D, Craig LC, Seaton A, Helms PJ, et al First trimester maternal tobacco smoking habits and fetal growth. *Thorax*. 2010;65:235–240. 10.1136/thx.2009.123232 20335293

[4] Bergsjo P, Bakketeig LS, Lindmark G. Maternal smoking does not affect fetal size as measured in the mid-second trimester. *Acta Obstet Gynecol Scand*. 2007;86:156–160. 10.1080/00016340600984696 17364277

[5] Bernstein IM, Plociennik K, Stahle S, Badger GJ, Secker-Walker R. Impact of maternal cigarette smoking on fetal growth and body composition. *Am J Obstet Gynecol*. 2000;183:883–886. 10.1067/mob.2000.109103 11035331

[6] Lampl M, Kuzawa CW, Jeanty P. Prenatal smoke exposure alters growth in limb proportions and head shape in the midgestation human fetus. *Am J Hum Biol*. 2003;15:533–546. 10.1002/ajhb.10140 12820195

[7] Alkandari F, Ellahi A, Aucott L, Devereux G, Turner S. Fetal ultrasound measurements and associations with postnatal outcomes in infancy and childhood: A systematic review of an emerging literature. *J Epidemiol Comm Health*. 2015;69:41–48. 10.1136/jech-2014-204091 25190820

[8] Guxens M, Ballester F, Espada M, Fernandez MF, Grimalt JO, Ibarluzea J, et al Cohort profile: The INMA—INfancia y medio ambiente—(environment and childhood) project. *Int J Epidemiol*. 2012;41:930–940. 10.1093/ije/dyr054 21471022

[9] Vik T, Jacobsen G, Vatten L, Bakketeig LS. Pre- and post-natal growth in children of women who smoked in pregnancy. *Early Hum Dev*. 1996;45:245–255. 8855398 10.1016/0378-3782(96)01735-5

[10] Zaren B, Lindmark G, Bakketeig L. Maternal smoking affects fetal growth more in the male fetus. *Paediatr Perinat Epidemiol*. 2000;14:118–126. 10791654 10.1046/j.1365-3016.2000.00247.x

[11] Pringle PJ, Geary MP, Rodeck CH, Kingdom JC, Kayamba-Kay's S, Hindmarsh PC. The influence of cigarette smoking on antenatal growth, birth size, and the insulin-like growth factor axis. *J Clin Endocrinol Metabol*. 2005;90:2556–2562. 10.1210/jc.2004-1674 15713720

[12] Hadlock FP, Harrist RB, Carpenter RJ, Deter RL, Park SK. Sonographic estimation of fetal weight. the value of femur length in addition to head and abdomen measurements. *Radiology*. 1984;150:535–540. 10.1148/radiology.150.2.6691115 6691115

[13] Iniguez C, Ballester F, Costa O, Murcia M, Souto A, Santa-Marina L, et al Maternal smoking during pregnancy and fetal biometry: The INMA mother and child cohort study. *Am J Epidemiol*. 2013;178:1067–1075. 10.1093/aje/kwt085 24008909

[14] Hanke W, Sobala W, Kalinka J. Environmental tobacco smoke exposure among pregnant women: Impact on fetal biometry at 20–24 weeks of gestation and newborn child's birth weight. *Int Arch Occup Environ Health*. 2004;77:47–52. 10.1007/s00420-003-0475-0 14593481

[15] Newnham JP, Patterson L, James I, Reid SE. Effects of maternal cigarette smoking on ultrasonic measurements of fetal growth and on doppler flow velocity waveforms. *Early Hum Dev*. 1990;24:23–36. 2265596 10.1016/0378-3782(90)90003-2

[16] Cochrane Library. Cochrane handbook for systematic reviews of interventions http://handbook.cochrane.org/chapter_9/9_5_2_identifying_and_measuring_heterogeneity.htm. Updated 2011. Accessed 02/16, 2016.

[17] Lindell G, Marsal K, Kallen K. Impact of maternal characteristics on fetal growth in the third trimester: A population-based study. *Ultrasound Obstet Gynecol*. 2012;40:680–687. 10.1002/uog.11125 22302307

[18] Vielwerth SE, Jensen RB, Larsen T, Greisen G. The impact of maternal smoking on fetal and infant growth. *Early Hum Dev*. 2007;83:491–495. 10.1016/j.earlhumdev.2006.09.010 17079098

[19] Iniguez C, Ballester F, Amoros R, Murcia M, Plana A, Rebagliato M. Active and passive smoking during pregnancy and ultrasound measures of fetal growth in a cohort of pregnant women. *J Epidemiol Comm Health*. 2012;66:563–570. 10.1136/jech.2010.116756 21228353

[20] Heil SH, Higgins ST, Bernstein IM, Solomon LJ, Rogers RE, Thomas CS, et al Effects of voucher-based incentives on abstinence from cigarette smoking and fetal growth among pregnant women. *Addiction*. 2008;103:1009–1018. 10.1111/j.1360-0443.2008.02237.x 18482424 PMC2731575

[21] Jeanty P, Cousaert E, de Maertelaer V, Cantraine F. Sonographic detection of smoking-related decreased fetal growth. *J Ultrasound Med*. 1987;6:13–18. 3546717 10.7863/jum.1987.6.1.13

[22] Anblagan D, Jones NW, Costigan C, Parker AJ, Allcock K, Coyne LH, et al Maternal smoking during pregnancy and fetal organ growth: A magnetic resonance imaging study. *PLoS ONE*. 2013;8:e67223 10.1371/journal.pone.0067223 23843995 PMC3700970

[23] Schmid M, Kasprian G, Marschalek J, Posch A, Balassy C, Prayer D. Maternal smoking and fetal lung volume-an in utero MRI investigation. *Prenat Diagn*. 2011;31:491–495. 10.1002/pd.2725 21351284

[24] Roza SJ, Verburg BO, Jaddoe VW, Hofman A, Mackenbach JP, Steegers EA, et al Effects of maternal smoking in pregnancy on prenatal brain development. The generation R study. *Eur J Neurosci*. 2007;25:611–617. 10.1111/j.1460-9568.2007.05393.x 17298594

[25] Taal HR, Geelhoed JJ, Steegers EA, Hofman A, Moll HA, Lequin M, et al Maternal smoking during pregnancy and kidney volume in the offspring: The generation R study. *Pediatr Nephrol*. 2011;26:1275–1283. 10.1007/s00467-011-1848-3 21617916 PMC3119805

[26] Whisner CM, Young BE, Pressman EK, Queenan RA, Cooper EM, O'Brien KO. Maternal diet but not gestational weight gain predicts central adiposity accretion in utero among pregnant adolescents. *Int J Obesity*. 2015;39:565–570. 10.1038/ijo.2014.202 25468827

[27] AlMakoshi A, Ellahi A, Sallout B, Devereux G, Turner S. Fetal growth trajectory and risk for eczema in a Saudi population. *Pediatric Allergy Immunol*. 2015;26:811–6. 10.1111/pai.12380 25817325

[28] Kfir M, Yevtushok L, Onishchenko S, Wertelecki, Bakhireva L, Chambers CD, et al Can prenatal ultrasound detect the effects of in-utero alcohol exposure? A pilot study. *Ultrasound Obstet Gynecol*. 2009;33:683–689. 10.1002/uog.6379 19444822 PMC3746738

[29] National Institute for Health and Care Excellence. How to stop smoking in pregnancy and following childbirth. https://www.nice.org.uk/guidance/ph26. Updated 2010. Accessed 01/06, 2016.

[30] Sterne JAC, Sutton AJ, Ioannidis JPA, Terrin N, Jones DR, Lau J, et al Recommendations for examining and interpreting funnel plot asymmetry in meta-analyses of randomised controlled trials. *Brit Med J*. 2011;343:4002. 10.1136/bmj.d4002 21784880

[31] Hill AB. The environment and disease: Association or causation? *Proc R Soc Med*. 1965;58:295–300. 14283879 10.1177/003591576505800503 PMC1898525

[32] Klebanoff MA, Levine RJ, Clemens JD, DerSimonian R, Wilkins DG. Serum cotinine concentration and self-reported smoking during pregnancy. *Am J Epidemiol*. 1998;148:259–262. 9690362 10.1093/oxfordjournals.aje.a009633

[33] Mook-Kanamori DO, Steegers EAP, Eilers PH, Raat H, Hofman A, Jaddoe VWV. Risk factors and outcomes associated with first-trimester fetal growth restriction. *J Am Med Assoc*. 2010;303:527–534. 10.1001/jama.2010.78 20145229

[34] Leonardi-Bee J, Smyth A, Britton J, Coleman T. Environmental tobacco smoke and fetal health: Systematic review and meta-analysis. *Arch Dis Child Fetal Neonatal Ed*. 2008;93:F351–61. 10.1136/adc.2007.133553 18218658

