# Supplementary material for: A systematic review of maternal smoking during pregnancy and fetal measurements with meta-analysis
Source: PLoS One. 2017 Feb 23;12(2):e0170946. doi: 10.1371/journal.pone.0170946 (PMC5322900; doi:10.1371/journal.pone.0170946)
Supplement: S1 Table — (DOCX) [file pone.0170946.s001.docx]

Table S1. Quality control analysis for the studies included in this review. Each domain is scored 1-3 (1=strong study design, 2=moderate and 3=weak) and studies with no weak domains are given a strong global score, as study with one weak domain has a moderate global study design score and >one weak domain leads to a study being classed as globally weak.

| **Study** | **Selection Bias** | **Study Design** | **Confounders** | **Blinding** | **Data Collection and Method** | **Withdrawals and Dropouts** | **Global Rating** |
| --- | --- | --- | --- | --- | --- | --- | --- |
| Sonographic detection of smoking-related decreased fetal growth. Jeanty 1987[1] | 3 | 2 | 3 | 2 | 2 | 3 | 3 |
| Effects of maternal cigarette smoking on ultrasonic measurements of fetal growth and on Doppler flow velocity waveforms. Newnham 1990 [2] | 3 | 2 | 3 | 2 | 2 | 3 | 3 |
| Pre and post natal growth in children of women who smoked in pregnancy  1996. Vik 1996[3] | 2 | 2 | 2 | 2 | 1 | 2 | 1 |
| Impact of maternal cigarette smoking on fetal growth and body composition. Bernstein 2000[4] | 3 | 2 | 2 | 2 | 2 | 2 | 2 |
| Maternal smoking affects fetal growth more in the male fetus. Zaren 2000[5] | 3 | 2 | 2 | 1 | 2 | 2 | 2 |
| Prenatal smoke exposure alters growth in limb proportions and head shape in the midgestation human fetus. Lampl 2003[6] | 3 | 2 | 2 | 2 | 2 | 1 | 2 |
| Environmental tobacco smoke exposure among pregnant women: Impact on fetal biometry at 20-24 weeks of gestation and newborn child’s birth weight. Hanke 2004[7] | 2 | 2 | 2 | 2 | 1 | 1 | 1 |
| The influence of cigarette smoking on antenatal growth, birth size and the insulin-like growth factor axis.Pringle 2005[8] | 2 | 2 | 3 | 2 | 2 | 1 | 2 |
| Maternal smoking and fetal growth characteristics in different periods of pregnancy. Jaddoe 2007[9] | 3 | 2 | 1 | 1 | 2 | 2 | 2 |
| Maternal smoking does not affect fetal size as measured in the mid-second trimester. Bergsjo 2007[10] | 3 | 2 | 3 | 1 | 2 | 1 | 3 |
| The impact of maternal smoking on fetal and infant growth. Veilwerth 2007[11] | 2 | 2 | 3 | 1 | 2 | 3 | 3 |
| Effects of voucher-based incentives on abstinence from cigarette smoking and fetal growth among pregnant women. Heil 2008[12] | 3 | 1 | 2 | 2 | 1 | 1 | 2 |
| First trimester maternal tobacco smoking habits and fetal growth. Prabhu 2010[13] | 3 | 2 | 1 | 1 | 2 | 2 | 2 |
| Active and passive smoking during pregnancy and ultrasound measures of fetal growth in a cohort of pregnant women. Iniguez 2012[14] | 3 | 2 | 1 | 1 | 1 | 1 | 2 |
| Impact of maternal characteristics on fetal growth in the third trimester: a population-based study. Lindell 2012[15] | 1 | 2 | 2 | 1 | 1 | 1 | 1 |
| Maternal smoking during pregnancy and fetal biometry. Iniguez 2013[16] | 3 | 2 | 1 | 1 | 1 | 1 | 2 |

**REFERENCES**

1.     Jeanty P, Cousaert E, de Maertelaer V, Cantraine F. Sonographic detection of smoking-related decreased fetal growth. *Journal of Ultrasound in Medicine* 1987; **6**(1): 13-8.

2.     Newnham JP, Patterson L, James I, Reid SE. Effects of maternal cigarette smoking on ultrasonic measurements of fetal growth and on Doppler flow velocity waveforms. *Early Hum Dev* 1990; **24**(1): 23-36.

3.     Vik T, Jacobsen G, Vatten L, Bakketeig LS. Pre- and post-natal growth in children of women who smoked in pregnancy. *Early Hum Dev* 1996; **45**(3): 245-55.

4.     Bernstein IM, Plociennik K, Stahle S, Badger GJ, Secker-Walker R. Impact of maternal cigarette smoking on fetal growth and body composition. *American Journal of Obstetrics & Gynecology* 2000; **183**(4): 883-6.

5.     Zaren B, Lindmark G, Bakketeig L. Maternal smoking affects fetal growth more in the male fetus. *Paediatr Perinat Epidemiol* 2000; **14**(2): 118-26.

6.     Lampl M, Kuzawa CW, Jeanty P. Prenatal smoke exposure alters growth in limb proportions and head shape in the midgestation human fetus. *Am J Hum Biol* 2003; **15**(4): 533-46.

7.     Hanke W, Sobala W, Kalinka J. Environmental tobacco smoke exposure among pregnant women: impact on fetal biometry at 20-24 weeks of gestation and newborn child's birth weight. *International Archives of Occupational & Environmental Health* 2004; **77**(1): 47-52.

8.     Pringle PJ, Geary MP, Rodeck CH, Kingdom JC, Kayamba-Kay's S, Hindmarsh PC. The influence of cigarette smoking on antenatal growth, birth size, and the insulin-like growth factor axis. *Journal of Clinical Endocrinology & Metabolism* 2005; **90**(5): 2556-62.

9.     Jaddoe VW, Verburg BO, de Ridder MA, et al. Maternal smoking and fetal growth characteristics in different periods of pregnancy: the generation R study. *Am J Epidemiol* 2007; **165**(10): 1207-15.

10.     Bergsjo P, Bakketeig LS, Lindmark G. Maternal smoking does not affect fetal size as measured in the mid-second trimester. *Acta Obstet Gynecol Scand* 2007; **86**(2): 156-60.

11.     Vielwerth SE, Jensen RB, Larsen T, Greisen G. The impact of maternal smoking on fetal and infant growth. *Early Hum Dev* 2007; **83**(8): 491-5.

12.     Heil SH, Higgins ST, Bernstein IM, et al. Effects of voucher-based incentives on abstinence from cigarette smoking and fetal growth among pregnant women. *Addiction* 2008; **103**(6): 1009-18.

13.     Prabhu N, Smith N, Campbell D, et al. First trimester maternal tobacco smoking habits and fetal growth. *Thorax* 2010; **65**(3): 235-40.

14.     Iniguez C, Ballester F, Amoros R, Murcia M, Plana A, Rebagliato M. Active and passive smoking during pregnancy and ultrasound measures of fetal growth in a cohort of pregnant women. *Journal of Epidemiology & Community Health* 2012; **66**(6): 563-70.

15.     Lindell G, Marsal K, Kallen K. Impact of maternal characteristics on fetal growth in the third trimester: a population-based study. *Ultrasound in Obstetrics & Gynecology* 2012; **40**(6): 680-7.

16.     Iniguez C, Ballester F, Costa O, et al. Maternal smoking during pregnancy and fetal biometry: the INMA Mother and Child Cohort Study. *Am J Epidemiol* 2013; **178**(7): 1067-75.
