# Supplementary material for: A systematic review of maternal smoking during pregnancy and fetal measurements with meta-analysis
Source: PLoS One. 2017 Feb 23;12(2):e0170946. doi: 10.1371/journal.pone.0170946 (PMC5322900; doi:10.1371/journal.pone.0170946)
Supplement: S2 Table — (DOC) [file pone.0170946.s002.doc]

Table S2. A summary of data in each of the studies included in this systematic review. INC=inclusion criteria. EXC=exclusion criteria. CPD=cigarettes per day. EFW=estimated fetal weight. BPD=biparietal diameter. HC=head circumference. FL=femur length. CRL=crown rump length. AC=abdominal circumference. MAD=mean abdominal diameter. Δ=change in.

| **Study** | **Exposure** | **Cohort name and inclusion/exclusion criteria** | **Sample size, gestation when measurement made** | **Outcome/effect size for a given gestation** | **Outcome/effect size for change in fetal measurement** |
| --- | --- | --- | --- | --- | --- |
| Sonographic detection of smoking-related decreased fetal growth  Jeanty 1987[1]  USA | Maternal smoking | INC: uncomplicated pregnancies; absence of congenital abnormality, normal fetuses at birth. EXC: teenage, drug-addicted or high risk pregnancies. | 952 pregnancies in cross-sectional (CS) study (gestation not stated). 40 in longitudinal study (L) investigated 6-24 (mean 15) times in pregnancy. BPD, AC FL, humerus length, recorded. | In CS study maternal smoking was not associated with reduced fetal measurements | In L study, BPD, FL and AC (but not EFW) were reduced, effect size not reported. |
| Effects of maternal cigarette smoking on ultrasonic measurements of fetal growth and on Doppler flow velocity waveforms.  Newnham 1990[2]  Australia | Maternal smoking | INC: singleton pregnancy; written consent. | 535 pregnancies. Scans at 18, 24, 28 and 34 wks. BPD, OCF, AC, and FL measured. | (non, reformed, <20, >20) Smoking => reduced BPD in males only at 24 wks (63.55 ± 0.44, 64.53 ± 0.79, 61.12 ± 0.55, 62.20 ± 0.91), 28 wks (75.95 ± 0.32, 76.48 ± 0.66, 74.74 ± 0.42, 75.73 ± 0.73) and 34 wks (89.79 ± 0.34, 90.96 ± 0.82, 88.29 ± 0.49, 90.25 ± 0.77). Reduced AC in males at 24 wks (205.13 ± 1.47, 209.45 ± 2.92, 199.83 ± 1.68, 200.60 ± 2.50)) and females at 34 wks (309.58 ± 1.45, 308.79 ± 2.55, 307.24 ± 2.01, 295.78 ± 6.73). | N/A |
| Pre and post natal growth in children of women who smoked in pregnancy  Vik 1996[3]  Norway and Sweden | Maternal smoking | INC: mothers attending centres in Sweden and Norway, para 1 or 2, recruited <20th gestational week | 530 pregnancies (including 185 smokers). Scans at 17, 25, 33 and 37 weeks. Focus on 17 and 35 week data. FL, MAD and BPD reported. | Small reductions associated with maternal smoking for MAD (105 vs 107mm) and FL (72.1 vs 71.5mm) at 37 but not 17 weeks | Longitudinal data only presented for post natal growth trajectories |
| Impact of maternal cigarette smoking on fetal growth and body composition.  Bernstein 2000[4]  USA | Maternal smoking | Cohort of women engaged in a smoking cessation study at the University of Vermont.  EXC: incomplete data. | 65 smoking mothers vs 36 non-smoking from a previous study.  Scans between 27 and 37 wks – each had 2 separated by 4 wks. HC, FL, AC, thigh muscle area, thigh fat area, and EFW measured. | N/A | Growth of AC and thigh muscle area reduced in smokers (AC 9.0 ± 0.3 mm/wk vs 10.3 ± 0.5 mm/wk (P = 0.01); thigh muscle area 64.1 ± 3.8 mm^2^/wk vs 76.4 ± 5.6 mm^2^/wk).  Growth of thigh fat content reduced in smokers (38 ± 3.7 mm^2^/wk vs 54.6 + 5.4 mm^2^/wk; P = .004) but no sig. dif. in fat at 33 to 37 wks - smokers had higher fat content at 27 to 32 wks.  Slower EFW growth smokers’ fetuses (171 ± 5.4 g/wk vs 193 ± 8.0 g/wk; P = .004). |
| Maternal smoking affects fetal growth more in the male fetus.  Zaren 2000[5]  Norway and Sweden | Maternal smoking | `SGA- Scandinavia' cohort data used.  INC: first visit before 20 weeks of pregnancy; speak Swedish or Norwegian.  EXC: gave birth before 37wks, missing ultrasound measurements. | 306 non-smoking mothers, 242 light smokers (1-9 cpd) and 308 heavy smokers (≥10 cpd).  Scans at (mean) week 17.3, 25.0, 32.9, and 37.0,). FL, BPD, and MAD measured. | Significantly smaller BPD in weeks 33 (mm 1-9 cpd -0.5 (p=0.018), ≥10 -0.6 (p=0.006)) and 37 (1-9 cpd -0.5 (p=0.047), ≥10 -0.6 (p=0.012)) and MAD in weeks 25 (1-9 cpd -0.6 (p=0.031), ≥10 -0.6 (p=0.011)), 33 (1-9 cpd -1.0 (p=0.007), ≥10 -1.4 (p=0.0001)) and 37 (1-9 cpd -1.8 (p=0.0002), ≥10 -2.1 (p=0.0001)) were found in smoke-exposed.  ≥10 cpd had BPD reductions in males only (wk18 43.53 vs 43.68 (SD 0.5); wk25 65.48 vs 66.11 (SD 2.1); wk33 87.69 vs 88.42 (SD 2.7); wk37 94.24 vs 95.13 (SD 2.8)). Female MAD affected earlier than males (wk 25 vs 33). | N/A |
| Prenatal smoke exposure alters growth in limb proportions and head shape in the midgestation human fetus.  Lampl 2003[6]  USA (Data from Belgium) | Maternal smoking | INC: provided informed consent, were hospital workers of middle class socioeconomic back- ground, and had unremarkable medical and pregnancy histories | 400 pregnancies. 34 longitudinal; 366 cross-sectional.  Longitudinal: Scans at 23 and 32 wks; 28 also had a scan at 27 wks – measured BPD, occipital-frontal diameter, Transverse abdominal diameter, Anterior-posterior abdominal  Diameter, and length of Humerus, Ulna, Femur and Tibia.  Cross-sectional: time of scan varied, but most at around 20 (n=128) and 32 (n=238) wks. Measured either FL and humerus length, or tibia, fibula, radius and ulna length. | Reduced BPD and abdominal diameter at 20 weeks. Reduced femur and fibula at 32 weeks. Magnitude not stated. | Between 23 and 27 weeks, greater incremental growth in occipito frontal circumference (OFC) and arm:leg ratio. By 32 weeks, exposure associated with reduced BPD/OFC ratio, arm and tibia length. Magnitude not stated. |
| Environmental tobacco smoke exposure among pregnant women: Impact on fetal biometry at 20-24 weeks of gestation and newborn child’s birth weight.  Hanke 2004[7]  Poland | Maternal smoking | INC: women at 20–24 wks who visited antenatal care units between April 1997 and April 1998.  EXC: chronic diseases; incomplete data; refused serum cotinine measurements. | 183 women.  Measured BPD, AC and FL between 20 and 24 weeks of gestation. | Regression coefficients by serum cotinine levels: BPD -0.495 (SE 0.175). In  serum cotinine concentrations below 10 ng/ml (i.e. environmental smoke exposure), BPD -0.172 (SD 0.091) (P = 0.06). | N/A. |
| The influence of cigarette smoking on antenatal growth, birth size and the insulin-like growth factor axis.  Pringle 2005[8]  UK | Maternal smoking | Data collected between April 1996 and July 1997.  INC: first prenatal visit before 20 wk, normal fetus; Caucasian mother. EXC: increased nuchal translucency, malformation, maternal steroid use; twin pregnancies. | 1650 pregnancies.  Scans at 20 and 30 wks. BPD, HC, AC and FL measured. | No difference at 20wks.  At 30 wks, FL and AC reduced with smoking. (non-smokers, stopped on pregnancy, <10 cpd, 10-20 cpd, >20 cpd) mm (SD): FL 62.3 (3.0), 62.0 (3.6), 61.6 (3.0), 61.2 (3.8), 61.1 (3.0). AC 288 (17), 284 (19), 283 (19), 285 (21), 286 (22). | N/A |
| Maternal smoking and fetal growth characteristics in different periods of pregnancy: The Generation R Study  Jaddoe 2006[9]  Netherlands | Maternal smoking | Generation R cohort.  All children were born between April 2002 and January 2006.  EXC: mothers who did not provide smoking info in 1st trim; twin pregnancies; fetal deaths; or missing birth outcomes. CRL to date up to 12wks 5days. | 7,098 pregnancies.  HC, AC, FL measured. Median gestational ages for the fetal ultrasound examinations were 13, 21 weeks and 30 weeks | Smoking not associated with HC or AC at 18-24 weeks, but smoking <5 cpd was associated with reduced FL (-0.12 z score [-0.23, -0.01]) and also smoking >9 cpd (z score = -0.37 [-0.55, -0.18]).  In late pregnancy: HC, AC and FL all affected. For >9cpd, -0.26 [-0.45, -0.08] HC, -0.25 [-0.43, -0.06] AC and -0.40 [-0.57, -0.22] FL. | Smoking until pregnancy known not associated with growth differences in HC or FL, but AC 0.35mm/wk [0.05, 0.65] higher.  Continued smoking associated with -0.56mm/wk [-0.73, -0.40] HC, -0.58mm/wk [-0.81, -0.34] AC, -0.19mm/wk [-0.23, -0.14] FL. |
| Maternal smoking does not affect fetal size as measured in the mid-second trimester.  Bergsjo 2007[10]  Norway and Sweden | Maternal smoking | Cohort from the areas of the University hospitals of Trondheim and Bergen (Norway) and Uppsala (Sweden).  INC: Caucasian women; spoke a Scandinavian language. | 561 women.  Scan at 17 wks; biparietal diameter [BPD], mean abdominal diameter [MAD] and femur length [FL] measured. | Fetal size not significantly affected by smoking at 17 wks. | N/A |
| The impact of maternal smoking on fetal and infant growth  Veilwerth 2007[11]  Denmark | Maternal smoking | Births between 1985-1987 (data from previous study used). Case only study design (all exposed) | 269 pregnancies included. BPD to date. Scans from wk 28 to delivery. EFW measured; FL to indicate fetal length. | N/A | >15 cpd => decreased 3rd trim growth velocity of weight, not length.  3rd trim growth ΔZ SCORE/28 days (SD): (0-15cpd; >15 cpd; Reduced (>15 at 18wks, 0-15 at 28wks); Continuous (>15 at 18 and 28 wks): Weight -0.031 (0.367); -0.097 (0.398); -0.068 (0.403); -0.178 (0.377). |
| Effects of voucher-based incentives on abstinence from cigarette smoking and fetal growth among pregnant women.  Heil 2008[12]  USA | Maternal smoking | INC: self-report smoking (even a puff in the past 7 days) at a prenatal visit; self-report an estimated gestational age of ≤20 weeks; reside within the county in which the study clinic is located; plan to remain in the geographical area for 6 months following delivery; and speaks English.  EXC: incarceration; having participated previously in the study; or residing currently with someone who participated in the study. | 77 women. Scans at 30 and 34 wks. Measured BPD, HC, FL, and AC; EFW and lean thigh area calculated.  Vouchers given to contingent on submitting breath specimens (first time) and then urine-cotinine levels ≤ 80 ng/ml; in non-contingent, vouchers given independent of smoking status. | N/A | Greater EFW growth in contingent (215.6 ± 9.1 g/wk) than non-contingent (177 ± 9.7 g/wk) conditions (P = 0.006). Fetal FL (0.20 ± 0.01 cm/wk vs 0.17 ± 0.01 cm/wk) and AC (1.15 ± 0.05 cm/wk vs 0.98 ± 0.06 cm/wk) also greater in contingent. In abstainers vs smokers, independent of group, greater increase in EFW (214.9 ± 13.0 versus 190.3 ±8.2). |
| First trimester maternal tobacco smoking habits and fetal growth  Prabhu 2010[13]  UK | Maternal smoking | Mothers recruited at first trimester scan between 1997 and 1999. Scan measurements retrieved retrospectively in 2007. | 1924 pregnancies.  CRL at 8-12 wks; FL and BPD at 18-22 wks. | No difference in CRL in smoking/non-smoking mothers. No difference in FL or BPD in mothers who smoked when first pregnant, but FL in mums smoking 12-30 cpd 0.91cm [0.07, 1.75] shorter than mums smoking 1-8 cpd at 18-22 wks. | NA |
| Active and passive smoking during pregnancy and ultrasound measures of fetal growth in a cohort of pregnant women  Iniguez 2012[14]  Spain | Passive maternal smoke exposure | INMA Cohort.  This study based in Valencia.  INC: at least 16 years of age, a singleton pregnancy, no chronic disease, no communication problems and not taken part in an assisted reproduction programme. | 780 pregnancies. Scans at 12, 20 and 32 wks; also 100 in wk 38. BPD, FL and AC measured => EFW. CRL to date. Hospital record access for scans between 10-41 wks. Urine cotinine at wk 32. | Passive smoking (ie exposure in restaurants) was associated with reduced BPD: % change: 20wks -4.1 [-7.7, -0.4], 32 wks -6.7 [-10.7, -2.8], 38 wks -4.8 [-7.5, -2.1].  Maternal smoking caused reductions in size of all parameters, most obvious at 32 and 38 wks. No obvious linear relationship. | Passive smoking associated with reduced growth in BPD (% change): 12-20 wks -5.8 [-9.5, -2.0], 20-32 wks -5.7 [-9.5. -1.8], 12-32 wks -7.2 [-11. -3.3], 12-38wks -5.2 [-7.8, -2.6].  Maternal smoking => reduced growth in all parameters, esp. in later wks. Not linear. |
| Lindell 201215 | Maternal smoking | INC: Singleton pregnancies  delivered Southern  Sweden between 1995 and 2009; estimated date of delivery  determined by US at 17–19 weeks; and second assessment of fetal size in the third trimester Delivery  at ≥37 weeks. | 56 792 pregnancies  Scan 17-19 and 32-34 weeks  EFW the outcome  Data linked to population registry | Mean EFW z score reduction at 32-34 weeks −0.14 (−0.16 to −0.12) comparing no smoking and 1-9 cpd, and also 1-9cpd and ≥10cpd | Mean reduction in weight (birth wt –EFW) was −0.14 [-0.12, -0.16] z scores per 7 weeks (−0.16 to −0.12) comparing no smoking and 1-9 cpd and ≥10cpd |
| Maternal smoking during pregnancy and fetal biometry  Iniguez 2013[16]  Spain | Maternal smoking | The INMA Mother and Child Cohort Study.  Recruited 2003 to 2008. INC: maternal age ≥ 16 years, singleton pregnancy, 10–13 weeks gestation at enrolment, unassisted conception, delivery scheduled at the reference hospital, no handicap in communication. | 2,478 pregnancies.  Scans at 12, 20 and 34 wks gestation. BPD, FL, AC and EFW reported.  Urine cotinine measured in 3rd trim in 2244. | Smoking not associated with growth parameters at 12 or 20 wks. At 34 weeks, % differences in SD score relative to non-smokers were:  Smokers at wk 12: EFW-7.7 [-11.8, -3.5], FL -9.4 [-13.4, -5.4], BPD -7.8 [-12.0, -3.5], AC -4.4 [-8.7, -0.1]. Smokers who quit by 12 weeks EFW −4.7 [-9.4, 0.0], FL -5.5 [-10.1, -0.9]. | % change in SD of growth in wks 20-34 relative to non smokers:  Smokers at wk 12: EFW -8.7 [-12.9, -4.5], FL -10.0 [-14.0, -5.9], BPD -8.0 [-12.3, -3.6], AC -4.8 [-9.1, -0.4].  Ex-smokers at wk 12): FL -6.0 [-10.7, -1.3]. |

References

1.     Jeanty P, Cousaert E, de Maertelaer V, Cantraine F. Sonographic detection of smoking-related decreased fetal growth. *Journal of Ultrasound in Medicine* 1987; **6**(1): 13-8.

2.     Newnham JP, Patterson L, James I, Reid SE. Effects of maternal cigarette smoking on ultrasonic measurements of fetal growth and on Doppler flow velocity waveforms. *Early Hum Dev* 1990; **24**(1): 23-36.

3.     Vik T, Jacobsen G, Vatten L, Bakketeig LS. Pre- and post-natal growth in children of women who smoked in pregnancy. *Early Hum Dev* 1996; **45**(3): 245-55.

4.     Bernstein IM, Plociennik K, Stahle S, Badger GJ, Secker-Walker R. Impact of maternal cigarette smoking on fetal growth and body composition. *American Journal of Obstetrics & Gynecology* 2000; **183**(4): 883-6.

5.     Zaren B, Lindmark G, Bakketeig L. Maternal smoking affects fetal growth more in the male fetus. *Paediatr Perinat Epidemiol* 2000; **14**(2): 118-26.

6.     Lampl M, Kuzawa CW, Jeanty P. Prenatal smoke exposure alters growth in limb proportions and head shape in the midgestation human fetus. *Am J Hum Biol* 2003; **15**(4): 533-46.

7.     Hanke W, Sobala W, Kalinka J. Environmental tobacco smoke exposure among pregnant women: impact on fetal biometry at 20-24 weeks of gestation and newborn child's birth weight. *International Archives of Occupational & Environmental Health* 2004; **77**(1): 47-52.

8.     Pringle PJ, Geary MP, Rodeck CH, Kingdom JC, Kayamba-Kay's S, Hindmarsh PC. The influence of cigarette smoking on antenatal growth, birth size, and the insulin-like growth factor axis. *Journal of Clinical Endocrinology & Metabolism* 2005; **90**(5): 2556-62.

9.     Jaddoe VW, Verburg BO, de Ridder MA, et al. Maternal smoking and fetal growth characteristics in different periods of pregnancy: the generation R study. *Am J Epidemiol* 2007; **165**(10): 1207-15.

10.     Bergsjo P, Bakketeig LS, Lindmark G. Maternal smoking does not affect fetal size as measured in the mid-second trimester. *Acta Obstet Gynecol Scand* 2007; **86**(2): 156-60.

11.     Vielwerth SE, Jensen RB, Larsen T, Greisen G. The impact of maternal smoking on fetal and infant growth. *Early Hum Dev* 2007; **83**(8): 491-5.

12.     Heil SH, Higgins ST, Bernstein IM, et al. Effects of voucher-based incentives on abstinence from cigarette smoking and fetal growth among pregnant women. *Addiction* 2008; **103**(6): 1009-18.

13.     Prabhu N, Smith N, Campbell D, et al. First trimester maternal tobacco smoking habits and fetal growth. *Thorax* 2010; **65**(3): 235-40.

14.     Iniguez C, Ballester F, Amoros R, Murcia M, Plana A, Rebagliato M. Active and passive smoking during pregnancy and ultrasound measures of fetal growth in a cohort of pregnant women. *Journal of Epidemiology & Community Health* 2012; **66**(6): 563-70.

15.     Lindell G, Marsal K, Kallen K. Impact of maternal characteristics on fetal growth in the third trimester: a population-based study. *Ultrasound in Obstetrics & Gynecology* 2012; **40**(6): 680-7.

16.     Iniguez C, Ballester F, Costa O, et al. Maternal smoking during pregnancy and fetal biometry: the INMA Mother and Child Cohort Study. *Am J Epidemiol* 2013; **178**(7): 1067-75.
