# Supplementary material for: A systematic review of maternal smoking during pregnancy and fetal measurements with meta-analysis
Source: PLoS One. 2017 Feb 23;12(2):e0170946. doi: 10.1371/journal.pone.0170946 (PMC5322900; doi:10.1371/journal.pone.0170946)
Supplement: S3 Table — (DOCX) [file pone.0170946.s003.docx]

Table S3. Results of the sensitivity analysis for second trimester measurements.

|  | Mean [95% CI] FL z score | Mean [95% CI] BPD z score |
| --- | --- | --- |
| Reduction in fetal measurement for the group exposed to high maternal cigarette consumption relative to the group with exposure to low consumption | - -0.03 - [-0.13, -0.06] - p=0.52 - 4 studies[1,2,3,4] - 977 high exposure - 1044 low exposure | - -0.09 - [-0.19, -0.00] - p=0.05 - 4 studies[1,2,3,4] - 1122 high exposure - 991 low exposure |
| Reduction in fetal measurement for the group whose mothers quit after becoming pregnant relative to the group whose mother were non-smokers at the start of pregnancy | - 0.00 - [-0.07, 0.07] - p=0.93 - 3 studies[1,2,3] - 890 quit - 6648 non smokers | - -0.05 - [-0.12, 0.01] - p=0.12 - 3 studies[1,2,3] - 897 quit - 6660 non smokers |
| Reduction in fetal measurement for the group whose mothers were ex-smokers before pregnancy relative to the group whose mothers were never smokers | - -0.02 - [-0.12, 0.07] - p=0.65 - 2 studies[5,1] - 555 ex-smoker - 2425 non smoker | - 0.05 - [-0.14, 0.24] - p=0.62 - 2 studies[5,1] - 565 ex-smoker - 2436 non smoker |

References

1.     Prabhu N, Smith N, Campbell D, et al. First trimester maternal tobacco smoking habits and fetal growth. *Thorax* 2010; **65**(3): 235-40.

2.     Jaddoe VW, Verburg BO, de Ridder MA, et al. Maternal smoking and fetal growth characteristics in different periods of pregnancy: the generation R study. *Am J Epidemiol* 2007; **165**(10): 1207-15.

3.     Pringle PJ, Geary MP, Rodeck CH, Kingdom JC, Kayamba-Kay's S, Hindmarsh PC. The influence of cigarette smoking on antenatal growth, birth size, and the insulin-like growth factor axis. *Journal of Clinical Endocrinology & Metabolism* 2005; **90**(5): 2556-62.

4.     Zaren B, Lindmark G, Bakketeig L. Maternal smoking affects fetal growth more in the male fetus. *Paediatr Perinat Epidemiol* 2000; **14**(2): 118-26.

5.     Iniguez C, Ballester F, Costa O, et al. Maternal smoking during pregnancy and fetal biometry: the INMA Mother and Child Cohort Study. *Am J Epidemiol* 2013; **178**(7): 1067-75.
