# Supplementary material for: A systematic review of maternal smoking during pregnancy and fetal measurements with meta-analysis
Source: PLoS One. 2017 Feb 23;12(2):e0170946. doi: 10.1371/journal.pone.0170946 (PMC5322900; doi:10.1371/journal.pone.0170946)
Supplement: S4 Table — *Data only available in one study. (DOCX) [file pone.0170946.s004.docx]

Table S4. Results of the sensitivity analyses for the third trimester. *Data only available in one study[^1^].

|  | Mean [95% CI] FL z score | Mean [95% CI] BPD/HC z score | Mean [95% CI] EFW z score | Mean [95% CI] AC/MAD z score |
| --- | --- | --- | --- | --- |
| Reduction in fetal measurement for the group exposed to high maternal cigarette consumption relative to the group with exposure to low consumption | - -0.17 - [-0.28, -0.06] - p=0.003 - 3 studies[2,3] - 632 high exposure - 808 low exposure | - -0.15 - [-0.26, -0.03] - p=0.01 - 3 studies[2,3] - 616 high exposure - 779 low exposure | - -0.06 - [-0.17, 0.04] - p=0.24 - 3 studies[2,3] - 637 high exposure - 780 low exposure | - -0.01 - [-0.12, 0.10] - p=0.84 - 3 studies[2,3] - 639 high exposure - 807 low exposure |
| Reduction in fetal measurement for the group whose mothers quit after becoming pregnant relative to the group whose mother were non-smokers at the start of pregnancy | - -0.01 - [-0.08, 0.07] - p=0.89 - 2 studies[3,4] - 743 quit - 5845 non smokers | - -0.00 - [-0.08, 0.08] - p=0.99 - 2 studies[3,4] - 721 quit - 5700 non smokers | - -0.00 - [-0.08,0.07] - p=0.93 - 2 studies[3,4] - 720 quit - 5687 non smokers | - -0.01 - [-0.09, 0.06] - p=0.77 - 2 studies[3,4] - 739 quit - 5800 non smokers |
| Reduction in fetal measurement for the group whose mothers were ex-smokers before pregnancy relative to the group whose mothers were never smokers | * | * | * | * |

References

1.     Iniguez C, Ballester F, Costa O, et al. Maternal smoking during pregnancy and fetal biometry: the INMA Mother and Child Cohort Study. *Am J Epidemiol* 2013; **178**(7): 1067-75.

2.     Zaren B, Lindmark G, Bakketeig L. Maternal smoking affects fetal growth more in the male fetus. *Paediatr Perinat Epidemiol* 2000; **14**(2): 118-26.

3.     Jaddoe VW, Verburg BO, de Ridder MA, et al. Maternal smoking and fetal growth characteristics in different periods of pregnancy: the generation R study. *Am J Epidemiol* 2007; **165**(10): 1207-15.

4.     Pringle PJ, Geary MP, Rodeck CH, Kingdom JC, Kayamba-Kay's S, Hindmarsh PC. The influence of cigarette smoking on antenatal growth, birth size, and the insulin-like growth factor axis. *Journal of Clinical Endocrinology & Metabolism* 2005; **90**(5): 2556-62.
