# Supplementary figures and images for: A systematic review of maternal smoking during pregnancy and fetal measurements with meta-analysis
Source: PLoS One. 2017 Feb 23;12(2):e0170946. doi: 10.1371/journal.pone.0170946 (PMC5322900; doi:10.1371/journal.pone.0170946)

Figure S1. A “print screen” showing details of the OVID literature search used in May 2016


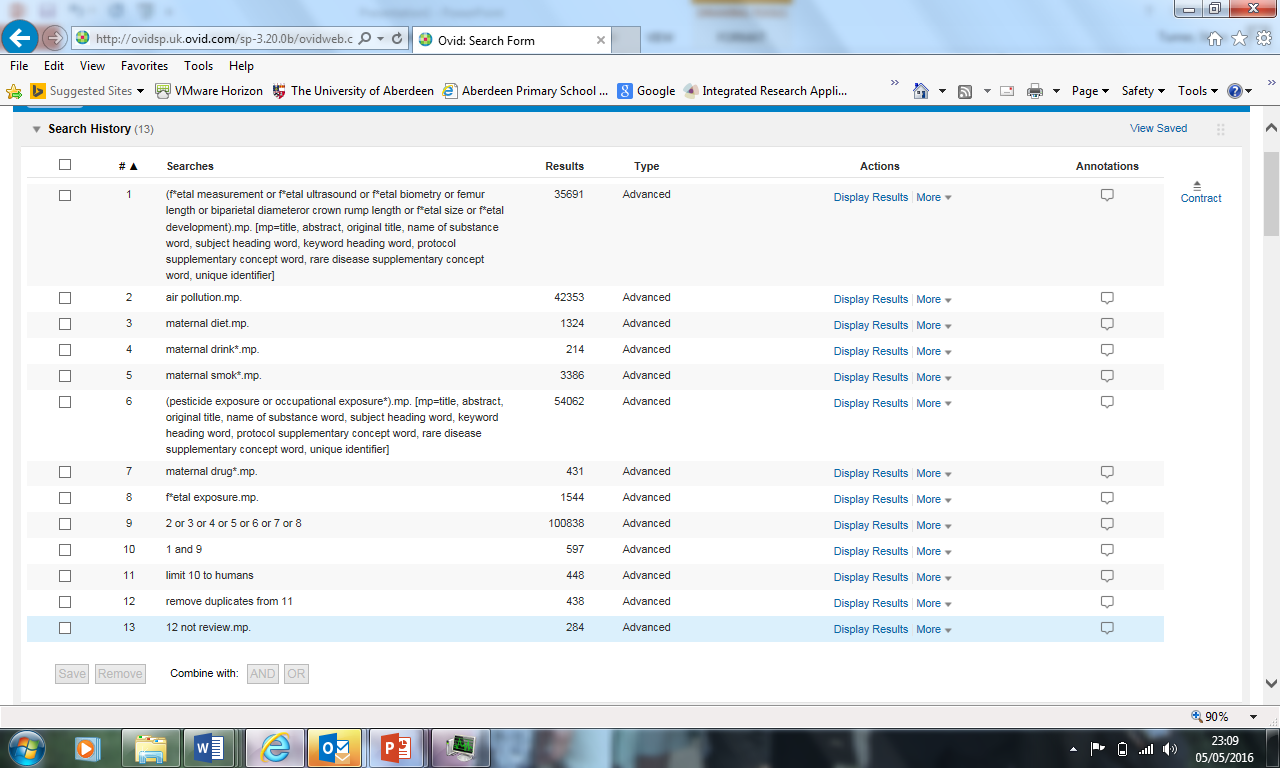

Supplement: S1 Fig — (DOCX) [file pone.0170946.s005.docx]
