# Supplementary material for: A systematic review of maternal smoking during pregnancy and fetal measurements with meta-analysis
Source: PLoS One. 2017 Feb 23;12(2):e0170946. doi: 10.1371/journal.pone.0170946 (PMC5322900; doi:10.1371/journal.pone.0170946)

Figure S4. Funnel plots for standardised second trimester measurements between individuals whose mothers smoked and did not smoke. The dashed sloping lines correspond to the area where 95% of studies are expected to lie assuming no bias or heterogeneity.

1. Biparietal Diameter


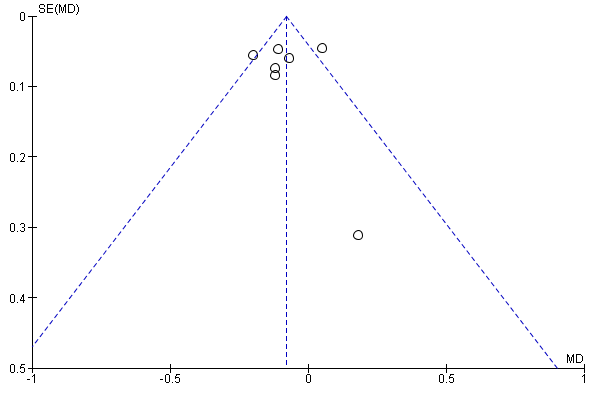


1. Femur Length


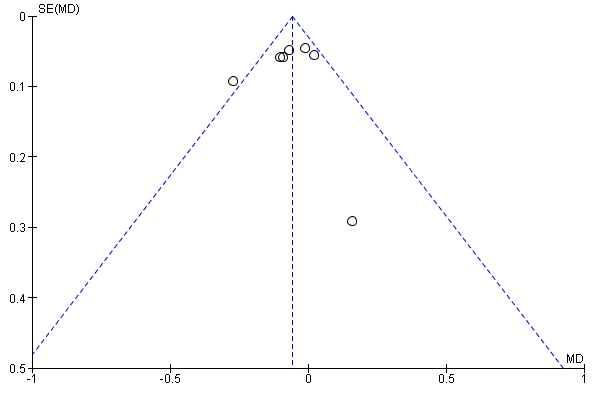


1. Abdominal Circumference


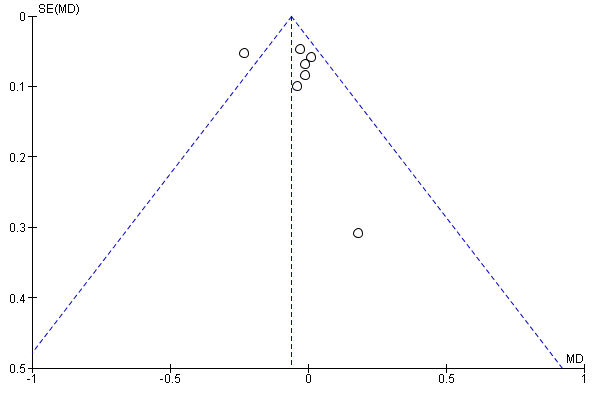


1. Estimated Fetal Weight


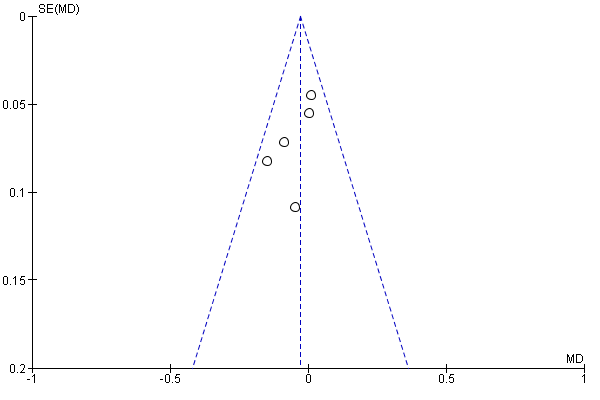

Supplement: S4 Fig — (DOCX) [file pone.0170946.s008.docx]
