# Supplementary material for: A systematic review of maternal smoking during pregnancy and fetal measurements with meta-analysis
Source: PLoS One. 2017 Feb 23;12(2):e0170946. doi: 10.1371/journal.pone.0170946 (PMC5322900; doi:10.1371/journal.pone.0170946)

Figure S6. Forest plots showing differences in absolute third trimester measurements between individuals whose mothers smoked and did not smoke

1. Biparietal Diameter


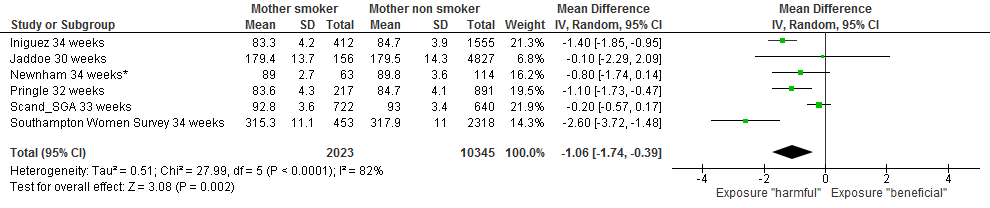


1. Femur Length


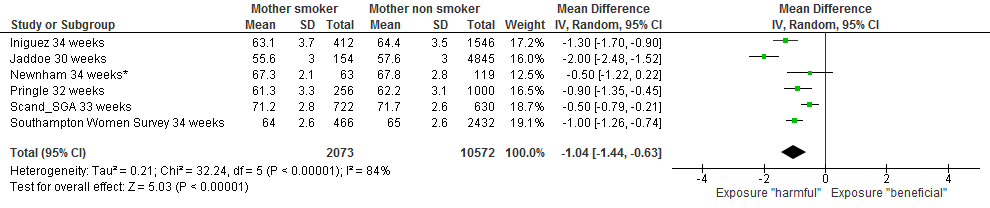


1. Abdominal Circumference


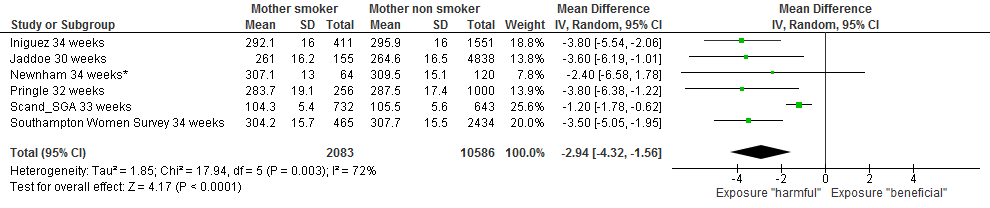


1. Estimated Fetal Weight


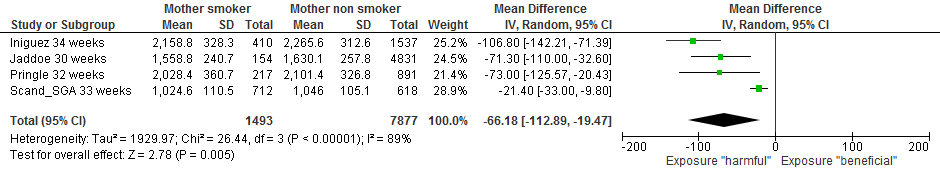

Supplement: S6 Fig — (DOCX) [file pone.0170946.s010.docx]
